# Combined ground and aerial measurements resolve vent-specific gas fluxes from a multi-vent volcano

**DOI:** 10.1038/s41467-020-16862-w

**Published:** 2020-06-16

**Authors:** T. D. Pering, E. J. Liu, K. Wood, T. C. Wilkes, A. Aiuppa, G. Tamburello, M. Bitetto, T. Richardson, A. J. S. McGonigle

**Affiliations:** 1 Department of Geography, University of Sheffield, Sheffield, S10 2TN UK; 2 Department of Earth Sciences, University College London, London, WC1E 6BS UK; 3 Department of Aerospace Engineering, University of Bristol, Bristol, BS8 1TR UK; 4 DiSTeM, Università di Palermo, via Archirafi, 36, 90123 Palermo, Italy; 5 Istituto Nazionale di Geofisica e Vulcanologia, Sezione di Bologna, Via Donato Creti, 12, 40128 Bologna, Italy; 6 School of Geosciences, the University of Sydney, Camperdown, NSW 2006 Australia; 7 Faculty of Health, Engineering and Sciences, University of Southern Queensland, Toowoomba, QLD 4350 Australia

**Keywords:** Natural hazards, Volcanology

## Abstract

Volcanoes with multiple summit vents present a methodological challenge for determining vent-specific gas emissions. Here, using a novel approach combining multiple ultraviolet cameras with synchronous aerial measurements, we calculate vent-specific gas compositions and fluxes for Stromboli volcano. Emissions from vent areas are spatially heterogeneous in composition and emission rate, with the central vent area dominating passive emissions, despite exhibiting the least explosive behaviour. Vents exhibiting Strombolian explosions emit low to negligible passive fluxes and are CO_2_-dominated, even during passive degassing. We propose a model for the conduit system based on contrasting rheological properties between vent areas. Our methodology has advantages for resolving contrasting outgassing dynamics given that measured bulk plume compositions are often intermediate between those of the distinct vent areas. We therefore emphasise the need for a vent-specific approach at multi-vent volcanoes and suggest that our approach could provide a transformative advance in volcano monitoring applications.

## Abstract (web summary)

Combining multiple ultraviolet cameras with synchronous aerial measurements, the authors here present vent-specific gas compositions and fluxes for Stromboli volcano. The results show that gas compositions vary between different vents, mirroring differences in eruptive behavior.

## Introduction

Explosive eruptions are driven largely by the rapid exsolution of magmatic volatiles (mainly H_2_O, CO_2_ and SO_2_), which exsolve from ascending and decompressing magmas at different depths corresponding to their respective solubilities in silicate melts^1–6^. Gas ratios measured in the emitted vapour phase at the surface thus provide direct constraints on the depth of degassing (i.e. equilibrium saturation pressure), and therefore directly inform our understanding of subsurface magmatic systems^5,7–11^. Abrupt changes in gas ratios, such as CO_2_/SO_2_, have been recorded prior to large eruptions^9,11–14^ or changes in the style of activity^15,16^, and therefore contribute to the forecasting of volcanic activity. Together with independent constraints on SO_2_ emission rates from remote sensing (UV/IR imaging or spectroscopy^17–25^); in situ measurements of gas composition enable quantification of total volatile emission rates from volcanoes^14,26–30^.

At volcanoes where there is only a single degassing vent, such as Villarrica (Chile) and Masaya (Nicaragua), procedures for determining SO_2_ emission rates and total volatile emissions are relatively well established. However, volcanoes with multiple degassing vents or fumaroles in close proximity present additional challenges, particularly if one or more vents exhibit explosive activity thereby restricting measurements to the crater rim^8,15,31,32^. At these multi-vent systems, differentiating between emissions from each vent or fumarole can be difficult due to a combination of rapid mixing between plumes and restricted viewing geometries for remote measurements. Commonly, therefore, only bulk plume measurements are recorded and the contribution of each vent to the overall degassing flux remains unconstrained. This uncertainty is particularly problematic when different vents are characterised by contrasting eruptive behaviours and degassing styles, and thus likely exhibit distinct gas signatures. Without vent-specific gas compositions and fluxes, critical information is lost that can inform not only our understanding of the subsurface magmatic system, but also the factors controlling variability in eruptive style.

The on-going development of Unoccupied Aerial System (UAS)-mounted gas sampling now enables in situ aerial measurements of gas concentrations in remote, high altitude or otherwise inaccessible volcanic plumes^30,33–37^ with increased spatial resolution and reduced costs/logistics compared to conventional aircraft surveys^14^. Until now, this UAS approach has yet to be integrated synchronously with the ground-based instrumentation needed to derive contemporaneous fluxes for all gas species in a multi-vent setting. The need for more spatially-resolved gas measurements is particularly important at persistently degassing volcanoes known to exhibit rapid shifts from passive to explosive degassing behaviour. Here, we present synchronous UV camera and aerial Multiple Gas Analyser System (Multi-GAS) measurements of degassing at the archetypal multi-vent volcano, Stromboli (Aeolian Islands, Sicily, Italy). By isolating gas compositions and emission rates from distinct degassing centres, and interpreting these data in the context of published constraints from other geophysical parameters, we contribute further insight into the shallow degassing processes and drivers of Strombolian explosions at this volcano.

## Results

### Stromboli volcano

**Fig. 1:**
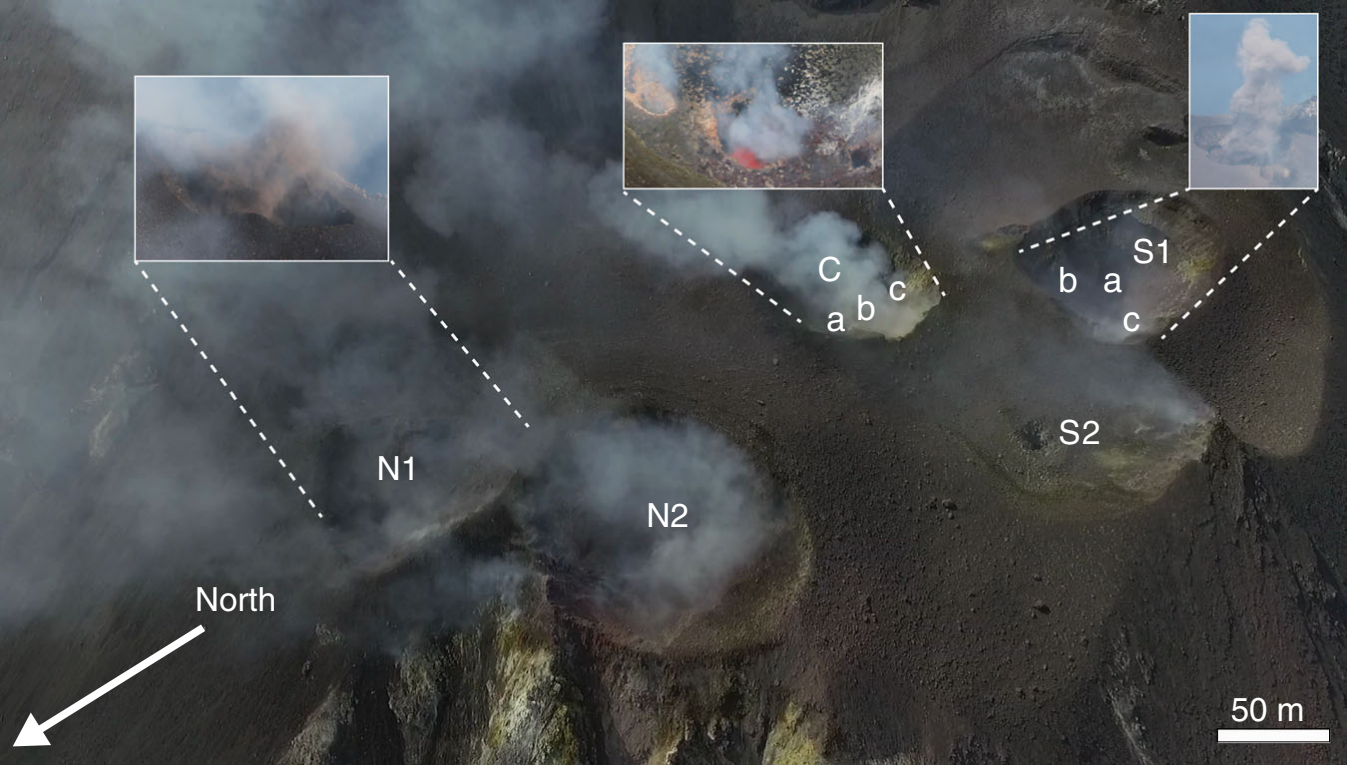
Stromboli volcano summit area. Multi-vent structure of the crater terrace of Stromboli, imaged during a UAS overflight on 6 June 2018. Indicated are main degassing areas (N Northern, C Central and S Southern) with numbers indicating separate vents. In response to the distinct eruptive behaviour and degassing characteristics observed, we retain these as separate vent areas rather than adopt the dual characterisation (N and SW + C) previously proposed. Lowercase letters indicate the location of individual intracrater vents where more than one is present. Inset are images of explosions from vents N1 and S1_a_, and incandescence and passive degassing within C_b_.

Stromboli is a composite multi-vent stratovolcano characterised by low viscosity shoshonitic basalts that enable effective gas-melt separation, and thus the persistent release of gases. The summit area of Stromboli is characterised by a broad crater terrace comprising multiple active vents. The number and spatial distribution of the three main degassing areas (see Fig. 1), and their eruptive characteristics, are dynamic and vary on timescales from hours-days to months–years^38–43^. On the basis of detailed analysis of jet dynamics and vent migration in the period 2005–2009, a dual categorisation of the crater terrace has been proposed, N and SW + C vent areas^42^.

Stromboli displays distinct modes of degassing: Passive degassing accounts for ~77%, while non-explosive active degassing (‘puffing’) and Strombolian explosions together account for ~23%^24,38,44^. All three degassing behaviours are often observed in parallel at different vents^32,38,39,42,44^. Strombolian explosions are short-duration, low to moderate intensity explosions, driven by the rapid expansion of Taylor bubbles (also known as gas slugs) and the subsequent impulsive ejection of gas and pyroclastic material^45–47^. Alternative models have also been proposed whereby gas slugs are formed within a crystal-rich plug^48,49^. Puffing is similarly associated with the rapid ejection of gases but from smaller Taylor or cap bubbles and therefore of lower intensity^38,39,41,50,51^. Passive degassing refers to the near-continuous non-explosive release of gases, again with no pyroclastic material. In addition to these three background behaviours, Stromboli occasionally exhibits major explosions and paroxysms of greater intensity, which generate pyroclastic flows and widespread tephra fallout^52,53^. Two major explosions occurred on 3 July 2019 and 28 August 2019, both preceded by lava efflux from all open vents (INGV Special Bulletins, for July and August); these were the largest explosions recorded since 2007. Given the popularity of Stromboli as a tourist destination, improving our understanding of what drives such dynamic evolution of vent behaviour, and particularly rapid passive to explosive transitions, is of critical importance for hazard assessment^54^.

### The summit area and eruptive activity

All vent areas (N, C and S) were active during our period of observation 4–12 June 2018 (Supplementary Movie 1). Here, because of the distinct eruptive behaviour and degassing characteristics observed, we retain these as separate vent areas rather than adopt the dual characterisation (N and SW + C) previously proposed (Fig. 1). The northern vent area (N) comprised two degassing centres; N1 and N2, which both exhibited Strombolian explosions several times every hour. Explosions from N1 were impulsive and energetic, and ejected ash-rich jets to heights of several hundred metres above the vent. No observable passive degassing occurred between explosions, indicating possible sealing of the magmatic column. In contrast, activity at N2 was dominated by continuous passive degassing with occasional low energy ash exhalations that reached only a few tens of metres above the vent before being dispersed downwind. Activity in the southern and central (S + C) vent area was focused at three main vents, which we refer to as C, S1 and S2. However, additional intracrater vents exhibiting low level passive degassing were visible within the main S1 and C depressions, and these are annotated on Fig. 1. Activity at S1 and S2 was dominated by Strombolian explosions occurring several times every hour, either at both vents simultaneously or at a single vent in isolation.

It was noted that no clear systematic relationship was evident in the relative timings of explosions from the N, S and C vent areas. Explosive activity at S1 included both ash-free gas exhalations and low to moderately energetic ash-rich explosions. Persistent inter-explosive passive degassing was visible from S1_a_, with unresolvable contributions from S1_b_ and S1_c_. A UAS overflight above S1 showed that during a typical ash-rich explosion, explosive gas release at one vent location preceded by ~1 s doming of the ground surface and pyroclast ejection at a second position several metres away (Supplementary Movie 1). Interestingly, this implies that a high proportion of the ash released during Strombolian explosions is derived from recycled material covering the vent area^55^ and has significant implications for Strombolian explosion dynamics^56,57^.

Explosions from S2 were comparatively energetic with rapid gas expulsion velocities, likely amplified by the small vent diameter of only a few metres, and contained no visible pyroclasts (including ash, lapilli or bombs). No passive degassing from S2 was detected between explosions. The central vent area (C) was persistently degassing during the period of observation, and provided the most sustained contribution to the bulk plume. Three discrete vents several metres in diameter (C_a–c_) were visible within the vent area, each characterised by regular puffing activity that often became stronger and more regular following an explosion at either S1 or S2 (Supplementary Movie 1). Incandescence and occasional spattering activity was observed at C_b_ on 7 June, during which time degassing was reduced or absent at the other two vents.

### SO_2_ emission rates

**Fig. 2:**
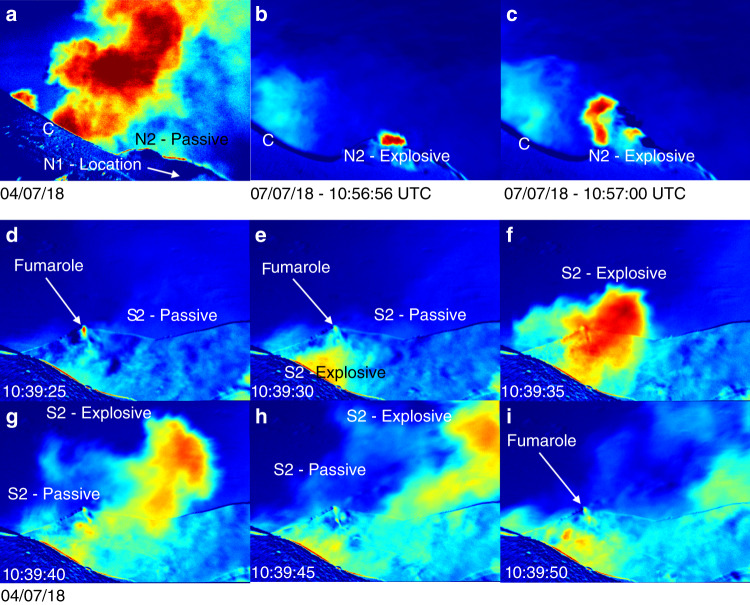
UV camera absorbance images. Example UV camera absorbance images showing activity from each individual vent imaged. **a** Data from 04/07/18 showing the high contributions of emissions from area C, lack of emissions from N1 and lower contribution to passive degassing from N2; **b**, **c** A minor Strombolian explosion from N2 on 07/07/18, as viewed from the north-east; **d**–**i** An image sequence showing the evolution of a Strombolian explosion from S2, as viewed from the south.

**Fig. 3:**
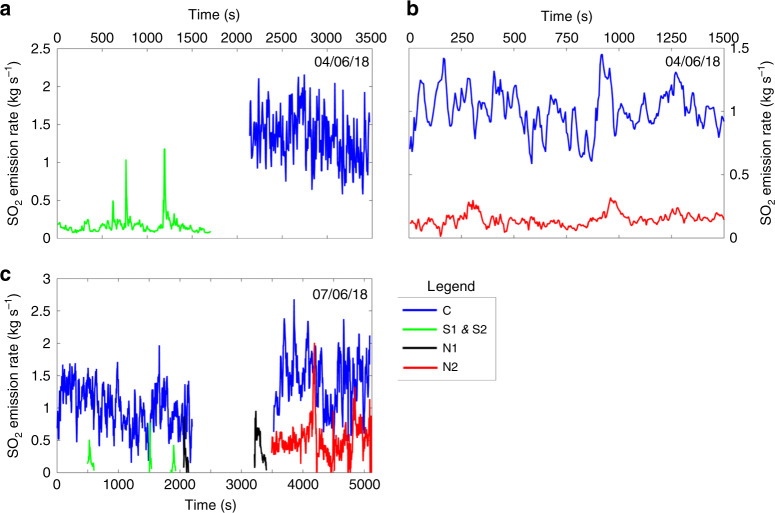
Vent-specific SO_2_ emissions. SO_2_ emissions from UV camera acquisitions on 04/06/18 and 07/06/18. In **a** two separate explosions from S1, and elevated emissions from area C; in **b** passive emissions from N2 and C and in **c** explosive emissions only from S1, S2 and N1, alongside passive emissions from C and combined passive and explosive emissions from N2. Note the difference in *y*-axis scale between plots.

UV camera measurements from targeted viewing angles allowed the determination of vent-specific SO_2_ emission rates from four vents (S1, S2, C and N2; see Fig. 2). We observed negligible SO_2_ from N1 (except during explosions, during which the acquisition was compromised by ash emission), suggesting that passive SO_2_ emissions were low and at most at the detection limit of the UV camera. Emission traces are presented in Fig. 3, with our measurements summarised in Table 1. Most of the SO_2_ emissions emanated from area C, which accounted for ~62–70% of total SO_2_ emissions (assuming that SO_2_ emissions from N1 are negligible). SO_2_ emissions on 04/06/18 are ~1.62–1.94 kg s^−1^ (~140–168 t d^−1^), and on 07/06/18 ~1.61–2.04 kg s^−1^ (~140– 180 t d^−1^), with RMS errors of ±13% on all values. Our measured SO_2_ emissions on 4 and 7 June are in the general range reported by the Flame network for the same period (Burton et al., 2015; and available via INGV weekly reports), which measured emission rates of ~130, 180 and 300 t d^−1^ SO_2_ on 3, 4 and 7 June, respectively, (no uncertainties given).

**Table 1:** Summary of vent-specific gas compositions and fluxes.

Vent	CO_2_/SO_2_	R^2^	*n* (s)	H_2_O/SO_2_	R^2^	*n* (s)	SO_2_ mol%	CO_2_ mol%	H_2_O mol%	SO_2_ (kg s^−1^)	CO_2_ (kg s^−1^)	H_2_O (kg s^−1^)	Total (kg s^−1^)
*Passive*													
S1	6.26 ± 0.76	0.82	230	22.8 ± 9.2	0.54	83	3.3	20.8	75.8	0.14 ± 0.02	0.6 ± 0.1	0.9 ± 0.14	1.6
C (FL1)	0.91 ± 0.14	0.65	345	32.2 ± 1.6	0.93	496	2.9	2.7	94.4	1–1.43 ± 0.2	0.63–0.89 ± 0.15	9.1–13.0 ± 2.1	10.7–15.3
C (FL2)	0.82 ± 0.16	0.66	236	34.5 ± 2.9	0.9	236	2.8	2.3	95.0	1–1.43 ± 0.2	0.56–0.81 ± 0.15	9.7–13.9 ± 2.7	11.3–16.1
C (average)							2.8	2.5	94.7	1.22 ± 0.2	0.72 ± 0.15	11.4 ± 2.4	13.3
N2 (FL1)	10.7 ± 1.9	0.75	169	19.6 ± 5.2	0.64	125	3.2	34.2	62.6	0.47 ± 0.06	3.46 ± 0.6	2.59 ± 1.3	6.5
N2 (FL2)	13.4 ± 2.3	0.71	214	22.2 ± 7.0	0.3	356	2.7	36.6	60.7	0.47 ± 0.06	4.94 ± 0.9	1.64 ± 1.7	7.1
N2 (average)							3.0	35.4	61.6	0.47 ± 0.06	4.2 ± 0.8	2.1 ± 1.5	6.8
*Explosive*													
N2 explosive (FL1)	15.3 ± 1.2	0.93	212	12.4 ± 2.7	0.73	212	3.48	53.31	43.21				
N2 explosive (FL2)	18.8 ± 2.1	0.95	71	23.2 ± 3.5	0.91	71	2.33	43.72	53.95				
N2 explosive (average)							2.90	48.52	48.58				

Fluxes were calculated by converting molar ratios to mass ratios and multiplying by the UV camera-derived SO_2_ fluxes. Bulk plume composition is an average composition measured over the duration of the campaign by the permanent Multi-GAS installation for monitoring.

### Gas molar ratios

**Fig. 4:**
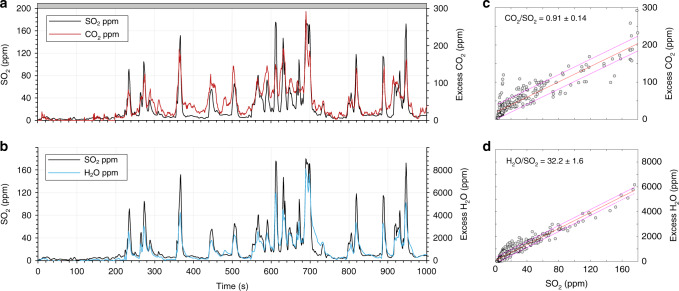
Molar gas compositions for UAS overflight on 07/06/18. Molar gas composition data for an UAS overflight on 07/06/18 during passive degassing from the central vent area (C), showing gas concentration timeseries for **a** SO_2_ and CO_2_, **b** SO_2_ and H_2_O and **c**, **d** the corresponding scatterplots, showing the least squares regression and 95% confidence intervals. Excess refers to the concentration of the gas species of interest above background, which was determined from clean air measurements made outside of the plume where SO_2_ = 0. Gas molar ratios correspond to the gradient of the regression line shown. The flight path comprised horizontal transects through the plume around 20 m downwind of the vent. Each peak indicates passage through the plume, with troughs indicating positions outside of the plume (e.g. where SO_2_ values = 0).

**Fig. 5:**
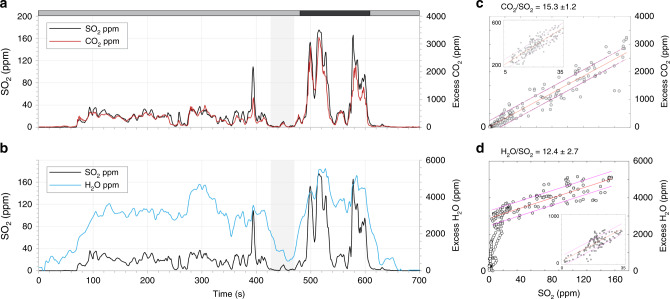
Molar gas compositions for UAS overflight on 10/06/18. Molar gas composition data for an UAS overflight on 10/06/18 during passive degassing and a Strombolian explosion from vent N2, showing gas concentration timeseries for **a** SO_2_ and CO_2_, **b** SO_2_ and H_2_O. The shaded band at the top indicates passive (grey) and explosive (black) phases. Note the drop in magmatic gas concentrations to background immediately prior to the first explosion (shaded column). **c**, **d** Scatterplots for the explosive phase emissions of SO_2_–CO_2_ and SO_2_–H_2_O, respectively, displaying the least squares regression and 95% confidence intervals. Excess refers to the concentration of the gas species of interest above background, which was determined from clean air measurements made outside of the plume where SO_2_ = 0. Gas molar ratios correspond to the gradient of the regression line shown. Note that for SO_2_–H_2_O, the regression is only based on SO_2_ > 10 ppm due to the highly variable H_2_O at low SO_2_ concentrations. Inset scatterplots show data for the passive degassing phase. See also Supplementary Fig. 2, for UAS overflight on 10/06/18 during passive degassing and a Strombolian explosion from vent N2.

**Fig. 6:**
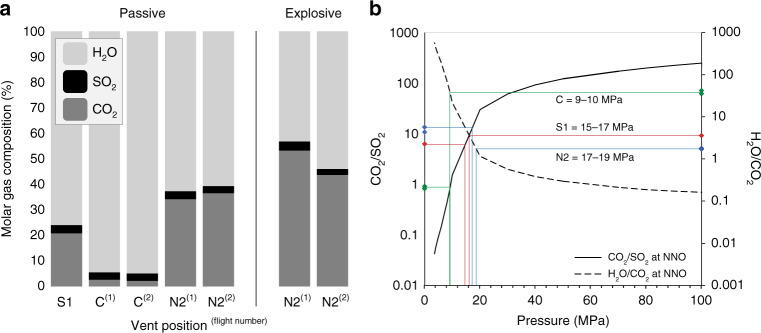
Vent-specific molar compositions and degassing pressures model. **a** Summary of vent-specific molar gas compositions during passive and explosive degassing. The elevated CO_2_ composition of N2 compared to C and S1 is evident. The composition of explosive gas emissions from N2 indicates elevated CO_2_ composition relative to the preceding and/or succeeding passive emissions in the same flight. **b** Degassing pressures: Measured passive/non-explosive gas compositions superposed on modelled gas compositions, expressed as CO_2_/SO_2_ (solid line) and H_2_O/CO_2_ (dashed line) during closed system degassing from 100 to 0.1 MPa buffered at NNO (nickel–nickel oxide redox buffer). See methods for details of model starting composition. Increasing equilibrium pressures (i.e. shallower depths of degassing) are observed from C to S1 to N2.

The concentrations of major volcanic gas species were measured in the emissions from three distinct vents: N2, C and S1. Data were acquired using an instrumented UAS equipped with a miniaturised Multi-GAS unit (see Methods) during either hovering (N2, S1 and C) or multiple lateral transects through the plume (C only). Sections of each timeseries were classified as either passive or explosive degassing based on visual observations made during each flight (Figs. 4 and 5). We find that molar gas ratios during non-explosive passive degassing (or puffing) vary considerably between the different vent areas but are consistent for a specific vent between multiple flights. H_2_S was not measured above the detection limit during any flight (see Methods). A summary of vent compositions is presented in Table 1 (see also Fig. 6, and Supplementary Figs.).

Overall, calculated molar compositions are distinct for each vent area (Fig. 6). Passive emissions from the central vent area (C) were dominated by water vapour (94–95 mol%) with <3% each of CO_2_ and SO_2_. In contrast, the vent area N2 was associated with a passive gas composition much richer in CO_2_ (34–37 mol%) and correspondingly reduced in H_2_O (61–63 mol%). Emissions of CO_2_ and H_2_O from the southern vent area (S1) were found to be intermediate between the two end members. Interestingly, the relative molar proportion of SO_2_ remained relatively constant (2.7–3.3 mol%) between all vent areas.

Vent proximal measurements were also acquired during Strombolian explosions, allowing direct comparison of the gas composition emitted during both passive and explosive degassing styles, and the transition between the two states (Fig. 5). Two explosions from N2 were intercepted from a measurement position approximately ten metres above the rim of the vent crater. The northerly wind direction at the time means we cannot exclude a passive degassing contribution from N1; however, the colocated UV camera indicated that N1 was releasing negligible passive emissions and so any signal would have been dwarfed by the explosive release from N2 (see section 2.2). The onset of each explosion was accompanied by an abrupt increase in the concentration of all gas species by a factor of 4–5 (see Fig. 5). Furthermore, in both cases the molar composition of the emitted vapour phase evolved to a more CO_2_-rich composition (up to 54 mol%) relative to the passive emissions measured immediately prior to or following the explosion. CO_2_/SO_2_ increased to 15.3–18.8 (±1–2) during explosive degassing (Table 1); however, the relative molar proportion of SO_2_ remained largely unchanged at 2.3–3.5 mol% due to the coincident reduction in H_2_O/SO_2_. Interestingly, in the 20 s prior to the explosion intercepted during the first flight over N2, the concentration of all gas species dropped to near-background atmospheric levels (see Fig. 5).

## Discussion

By combining vent-specific SO_2_ emission rates with aerial molar gas ratios measured during passive degassing (and puffing), we have quantified multispecies gas fluxes for each vent area (Table 1). Overall, we estimate that the total outgassing volatile budget from all vent areas is ~21.9 kg s^−1^ (1890 t d^−1^), of which SO_2_, CO_2_ and H_2_O contribute ~1.8 kg s^−1^ (~160 t d^−1^), ~5.5 kg s^−1^ (~480 t d^−1^) and ~14.4 kg s^−1^ (~1240 t d^−1^), respectively. Of this total, most of the gas (~61% by mass; [~13.3 kg s^−1^, ~1150 t d^−1^]) is derived from the central vent area, which is predominantly composed of H_2_O (11.4 kg s^−1^, 980 t d^−1^). In contrast, the volatile fluxes from N and S vent areas are significantly lower (31% by mass [~6.8 kg s^−1^, ~550 t d^−1^] and 7% by mass [~1.6 kg s^−1^, 140 t d^−1^], respectively). However, these areas emit proportionally more CO_2_, with 38% by mass CO_2_ (0.6 kg s^−1^, 50 t d^−1^) in the S vent area, and 73% by mass CO_2_ (4.9 kg s^−1^, 420 t d^−1^) in the N vent area. Note that switching of the locus of Strombolian explosions between vent areas is common, and the central vent area can exhibit more explosive activity than during our observation period^39,42,44^.

A key observation from these flux data is that the central vent area is by far the dominant emission source by mass during passive outgassing, despite exhibiting the least explosive behaviour. Sustained puffing activity from multiple vents, combined with visible incandescence, indicates effective gas-melt separation at shallow depths within this vent area. The higher proportions of CO_2_ combined with markedly lower passive fluxes from N and S vent areas are therefore interesting from the perspective of understanding eruption mechanisms, as these are the two craters from which Strombolian explosions place. Our data suggest relatively little open-system gas release between explosions, particularly from N2, supporting previous suggestions of a rheologically stiffened low permeability cap in the uppermost conduit^48,49,56,58^. If the passive volatile flux can be taken as a proxy for the relative permeability of the shallow conduit between vent areas, this indicates that the central vent area retains high permeability throughout. In contrast, N and S vent areas display outgassing behaviours consistent with a variably-degassed crystallisated magma cap that modulates short-timescale rheological variations, with implications for periodic overpressure development. Our results are even more intriguing when it is considered that major explosions most commonly occur from the central vent area^59^, as this highlights that some fundamental change from this background regime must take place prior to or during these large events.

We observe a near-complete cessation in magmatic gas emissions ~20–30 seconds prior to an explosion (Fig. 5), during which the concentration of SO_2_ (the plume tracer species) drops to zero. We note that although the excess H_2_O concurrently decreases, it remains slightly elevated at ~400 ppm above background, and we attribute this to the combined effect of natural variability in the ambient background humidity and a slower sensor response. Nevertheless, this abrupt reduction in emissions provides an intriguing indication that more efficient conduit sealing may precede the onset of an explosion, through a rapid reduction in magma permeability^60,61^. Although we cannot exclude the possibility that the UAS moved temporarily out of the plume due to a shift in wind direction during this interval, we did not observe any noticeable meteorological change to this effect in either our line-of-sight observations from the crater rim or in the first-person camera view from the UAS. Indeed, a similar but less pronounced reduction in emissions was observed prior to a second explosion at N2 (Supplementary Fig. 2). We highlight the need for further aerial measurements to explore in more detail this mechanism for passive to explosive transitions.

**Fig. 7:**
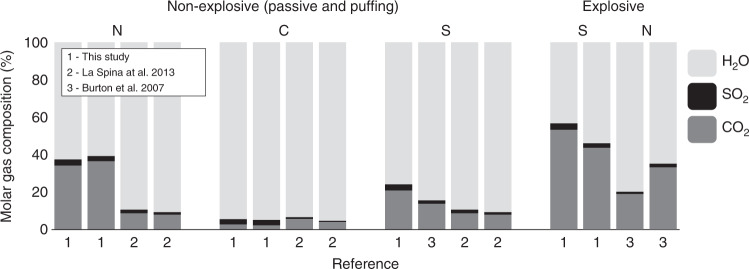
Comparison of molar gas compositions with previous studies. Comparison of molar gas compositions (mol %) of major species measured in this study, to previously published data for both non-explosive (passive degassing/puffing) and explosive degassing.

The molar gas compositions measured in this campaign fall within the ranges of previous studies that differentiated between emissions from distinct vent areas^58,62^, but with some notable differences (Fig. 7). For example, at vent area N, we measured substantially elevated molar proportions of CO_2_ during non-explosive degassing than previously described (34–37 mol% compared to 8–9 mol%^58^). Crucially, our results provide further support to the hypothesis that gas emissions during transient explosions are more CO_2_-rich than those from continuous passive degassing. We find that that molar proportion of CO_2_ increases at the northern vent area by 24–50 mol% relative to passive emissions, consistent with the lower end of the 40–143 mol% increase previously documented at the southern vent area^62^. These temporal differences emphasise both the dynamic nature of the volcanic activity at Stromboli, and also the necessity of vent-specific analysis to track spatial heterogeneity through time. However, the reproducibility of these passive-explosive characteristics lends great support to process-based models of Strombolian explosions built around the principle of deeply sourced CO_2_-rich gas^63^.

The gas-melt partitioning behaviour of major magmatic volatile species is strongly pressure-dependent, and so the composition of the vapour phases can be related thermodynamically to the pressure (i.e. depth) of gas-melt segregation and final equilibration^2,5,13,62^. The equilibrium gas composition of surface emissions can therefore be simulated based on thermodynamic models of volatile solubility, calibrated on experimental data^2–4,6^. Here, we simulate the evolution in gas composition as a function of pressure from 100 to 0.1 MPa under conditions of closed system degassing using the model presented by Moretti and Papale^4^. From a shoshonitic (potassic, iron-poor and near silica-saturated^64^); starting melt composition (based on melt inclusion compositions from Métrich et al.^65^ and the same initial conditions as Aiuppa et al.^8^) and buffered redox conditions of either NNO (where NNO refers to the nickel–nickel oxide redox buffer), we compare our aerial measurements to modelled CO_2_/SO_2_ and H_2_O/CO_2_ ratios in the gas phase in order to estimate the pressure of gas-melt segregation (Fig. 6b). Full details of the model are presented by Aiuppa et al.^8^.

Figure 6b illustrates how the pressure of gas-melt equilibration (i.e. depth of degassing), under closed-system conditions, increases from C to S1 to N2. While active ‘puffing’ emissions from the central vent area (C) reflect relatively low pressure degassing of ~9 MPa, the higher CO_2_/SO_2_ ratios characteristic of the southern (S1) and northern (N2) vent areas are consistent with greater equilibration pressures (P_eq_) of 15–17 and 17–19 MPa, respectively. Under a lithostatic pressure gradient, these P_eq_ correspond to approximate depths of ~330 m (C), ~550–625 m (S1), and ~625–700 m (N2) beneath the surface. When the uncertainties on the molar gas ratios are considered, P_eq_ ranges overlap for S1 and N2 and therefore they cannot be resolved fully. We also note that these depths are underestimates for the case of an established conduit, and that a magmastatic pressure gradient (assuming the density of vesicular magma to be 1.5 g cm^−3^) yielding ~600 m (C), ~1000–1150 m (S1) and ~1150–1300 m (N2) may be a more appropriate upper bound. Explosions from N2 reflect yet further elevated degassing pressures of 19–24 MPa, which are at the lower limit of the 20–50 MPa range for ‘small explosions’ proposed in previous literature, consistent with visual observations of explosion magnitude^62^. We note that the choice of solubility model, starting melt composition and redox condition has a large effect on the absolute equilibration pressure, therefore we urge caution against over-interpreting absolute pressures. Nevertheless, we consider the relative differences in depth of gas-melt separation between vents to be robust. Together, these data suggest that the depth of final gas-melt equilibration is most shallow in the central vent area (more H_2_O-rich emissions), and deeper in the N and S vent areas (more CO_2_-rich emissions). Alternatively, if we interpret our data in the context of multiple gas sources^8,32^, then the different gas ratios measured at the surface instead populate a mixing trend between a deep CO_2_-rich vapour phase and SO_2_- and H_2_O-rich vapours derived from degassing within the shallow conduit system. The elevated CO_2_/SO_2_ ratios measured at S1 and N2 would then imply a greater connection to the deeper gas source, with little re-equilibration during low pressure ascent through the shallow magmatic system as a segregated vapour phase. In contrast, puffing from the central vent area would be driven predominantly by low pressure degassing from magma within a shallow reservoir, consistent with previous data indicating an H_2_O-rich gas composition^32^ and elevated magma temperatures^39^.

Combining modelled equilibration pressures with vent-specific gas fluxes, we propose a conceptual model to explain the link between the observed outgassing dynamics and the subsurface plumbing system. At the central vent area, the stable ‘puffing’ activity and elevated volatile fluxes (relative to other vent areas) during passive emissions require that the shallow conduit remains highly permeable to gas escape. Further, solubility constraints show that the sustained gas supply required to maintain the observed outgassing rates must derive from low pressure degassing, which is best explained by late-stage gas-melt separation during shallow magma convection. If indeed the main ‘branch’ of the subsurface conduit system lies beneath central vent area, as previously suggested^39^, elevated magma temperature may provide an important feedback that inhibits crystallisation and thus maintains permeability^32^ aided by increased throughput of higher volumes of gas. In contrast, the modest to negligible passive fluxes from N and S vent areas between Strombolian explosions suggest reduced permeability in the shallow conduits. Together with more CO_2_-rich passive emissions (compared to central vent area), these observations point to a deeper gas-melt separation and a lack of shallow magma replenishment. In the context of a branched conduit system, lateral vents with reduced flow, and thus lower magma temperatures, can develop plug-like rheologies. The proposed rheological contrast between C and N + S vent areas can explain the spatial distribution of explosive activity as, according to the crystal-rich plug models for Strombolian explosions^48^, rheological factors such as reduced permeability and increased bulk viscosity are critical to overpressure. Further, the greater saturation pressures calculated for explosive emissions are consistent with those studies advocating for a deep origin for the gas slugs that drive Strombolian activity^62^.

**Fig. 8:**
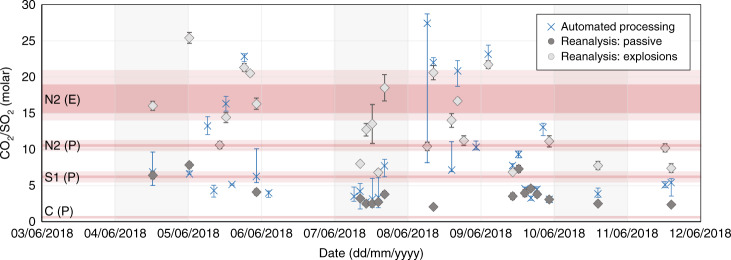
A comparison of ground based on UAS CO_2_/SO_2_ molar ratios. Ground-based multi-GAS measurements from the permanent monitoring station located on the crater rim of Stromboli. Timeseries of CO_2_/SO_2_ molar gas ratios measured 4–11 June 2018, showing automatically processed (blue crosses) and reanalysed (grey symbols) ratios. Passive degassing (dark grey symbols) represents persistent activity measured over tens of minutes, while explosive degassing (light grey symbols) represents transient peaks lasting <60 s. Red shaded bars correspond the aerial vent-specific gas compositions and 95% confidence intervals presented in Table 1 for passive (P) and explosive (E) emissions.

Ground-based multi-GAS measurements from the permanent monitoring station on the crater rim^8,12^ during 4–11 June 2018, highlight the temporal variability of bulk plume compositions (Fig. 8, and Supplementary Fig. 4). CO_2_/SO_2_ (molar) during passive degassing, defined as persistent activity measured over tens of minutes, spanned 2.1–7.8 with a stable average composition of 3.9 ± 1.4 (σ). These molar ratios are intermediate between our measured values for C, S2 and N2 vent areas, demonstrating that variable amounts of plume mixing must occur during transport to the crater rim. Passive emissions measured on the crater rim are therefore ‘bulk’ plume measurements. CO_2_/SO_2_ molar ratios during explosive degassing, defined as transient peaks in the timeseries lasting <60 s, span 6.8–25.4 with an average composition of 14.0 ± 4.35 (σ). This composition is in good agreement with the elevated ratios (CO_2_/SO_2_ = 15.3 ± 1.2 and 18.8 ± 2.1) derived from aerial measurements during explosive activity at N2 (Fig. 5). The more CO_2_-rich compositions in the monitoring timeseries (up to CO_2_/SO_2_ = 25) may derive from explosive exhalations from N1, which at the time of our measurements was exhibiting significantly more energetic Strombolian explosions than N2 and therefore could not be characterised safely using the UAS. CO_2_/SO_2_ has been shown to scale with explosion magnitude^62^.

The challenges of using a single Multi-GAS station in a multi-vent context are highlighted by Fig. 8. Calculated ratios are hybridized values based on contributions from multiple vents and contrasting degassing styles, but this value is strongly dependent on the prevailing wind direction as this determines the nature of plume mixing^58^. For example, the wind direction on 7 June was westerly, resulting in strong passive contributions from C and S1 and a low average CO_2_/SO_2_ ratio of 2.9 ± 0.6. In comparison, the south-easterly wind on 8 June meant that passive degassing was generally below detection and only strong explosive emissions reached the station, yielding a high average CO_2_/SO_2_ of 14.6 ± 1.7. Bulk plume molar proportions of 48–98 mol% (mean, 80%), 2–50 mol% (mean, 17%) and 0.2–14 mol% (mean, 3%) have been presented previously for H_2_O, CO_2_ and SO_2_, respectively^8^. Our data, based on vent-specific measurements, are within the quoted ranges for all vents; however, we emphasise that the bulk composition is not wholly representative of degassing at any single vent. Distinguishing between differences in the relative contributions of distinct vent-specific emissions from true changes in the subsurface conditions of degassing is critical to the use of gas measurements as a robust monitoring parameter.

We show, using a novel approach combining ground-based UV remote sensing of SO_2_ flux with vent-specific aerial measurements of gas composition, that volcanic gas emissions at Stromboli are spatially heterogeneous in both chemistry and emission rate between distinct vent areas. The central vent area (C) contributed 61% of the total emissions, and therefore dominated the emissions budget. Emissions from vent area C were non-explosive, H_2_O-rich, characterised by 95 mol% H_2_O and H_2_O/SO_2_ ratios of 32–35, and associated with puffing. Contrastingly, the southern (S) and northern (N) vent areas exhibited frequent Strombolian explosions, with passive emissions characterised by greater proportions of CO_2_ (35 mol %) and lower overall volatile fluxes. To explain our observations and modelled degassing depths (shallowing from C to S1 to N2), we propose a conceptual model whereby the gas-rich central vent area is sustained by shallow convection and associated late-stage separation between gas and melt, which maintains a high permeability in the shallow conduit. In contrast, the gas-poor but proportionally CO_2_ rich S and N vent areas show evidence for reduced permeability to a deeply sourced gas, a condition that would favour the formation of a crystal-rich plug and thus promote Strombolian activity. By comparing vent-specific measurements to ‘bulk plume’ estimates from a permanent monitoring installation, we highlight that variable degrees of plume mixing during transport to the crater rim generate bulk plume signatures that are intermediate between the molar compositions characteristic of the distinct vent areas. Therefore, sub-daily to daily variations in CO_2_/SO_2_ may result simply from changes in the relative contribution of different vents (e.g. by a change of wind direction). This spatial heterogeneity has implications for the interpretation of monitoring data based on bulk plume measurements and emphasises the need for a vent-specific approach, using our novel combined approach, at multi-vent volcanoes to understand further the links between degassing and eruptive style.

## Methods

### Ultraviolet camera

UV camera data were collected using the low-cost ‘PiCam’ on 04/06/18 and 07/06/18, totalling ~50 min on 04/06/18 and ~3 h on 07/06/18. Here, we used a modified system setup to that described in Wilkes et al., 2017, 2016^25,66^, by incorporating ‘PiJuice’ hardware and software (https://github.com/PiSupply/PiJuice) we were able to reduce the dimensions of the equipment, significantly increasing portability and potential for long-term remote placement. The PiJuice is an add-on for the Raspberry Pi micro-computer (https://www.raspberrypi.org/), which enables uninterruptible power supply to the Raspberry Pi via mobile phone batteries (we tested 1600 mAh and 2300 mAh batteries), which were recharged in-the-field using 40 W solar panels, i.e. one PiJuice and 40 W solar panel for each Raspberry Pi (see Supplementary Fig. 5). On their own, the phone batteries 2300 mAh batteries provided ~2 h of acquisition time. Each camera also incorporated a visible camera, attached to the ‘Raspberry Pi Zero’ micro-computer, which was powered using the GPIO (General Purpose Input/Output) pins of one of the PiJuice units, with colour image stills captured every second. This setup, with sufficient solar exposure, allowed the continued use of the cameras without the need for a USB power bank as per refs. ^25,66^.

Cameras were deployed around the summit area (see Supplementary Fig. 5), to enable the isolation of SO_2_ emission rates from each vent (see Fig. 4). Each camera had two Edmund Optics Inc. filters, centred at 310 and 330 nm, which account for where SO_2_ does and does not absorb, respectively. These filters had full width at half maximum of 10 nm. Images were captured at acquisition rates of 0.2–0.25 Hz, dependent on light conditions, and UTC GPS time-stamped. After acquisition, images were aligned, and dark and clear image corrected to account for the effects of sensor noise and vignetting respectively. Using the Beer–Lambert law pixel intensities were converted to column amounts using gas calibration cells (205, 1022, 1960 ppm m; with manufacturer quoted errors of ~10%). At certain camera locations, gas cell calibration was performed using a uniform ocean background, producing acceptable calibration lines (R^2^ > 0.98 in each instance), elsewhere, clear sky was used.

Plume speeds were high (>4 m s^−1^), therefore cross-correlation was used as optical flow did not efficiently track individual pulses of gas given proximity to the plume, see Fig. 4d–i. In addition, light conditions did not allow faster camera acquisition rates. SO_2_ emission rates were then determined through multiplication of plume speed by integrated column amounts (ICA) along a defined line in each image. Each ICA was chosen to isolate individual vent emissions, and were also used to remove the emissions of one vent from others, when plumes from two sources combined. All camera locations are provided in supplementary Fig. 5.

Overall, root-mean-square errors are estimated at ~13%. These are based on: low-light dilution effect given proximity to plume ~20%, with the lower of the 20–80% indicated by ref. ^67^ used here; the plume was not optically thick, ergo deviation from the Beer–Lambert law were deemed to be low;^68^ plume speed errors of ±10%^69,70^; manufacturer quoted ~10% errors in gas calibration cells; and finally error associated with distance to the plume. The latter error was minimised through use of UAS data, which pinpointed plume direction and hence position at integration line, and through manual estimates of plume direction in-the-field and within the UV camera imagery. We estimate that our inference of plume distance, based on the above estimates, are therefore low, however, a deviation of ±20 m, could lead to errors of 10% in SO_2_ flus values at a distance of ~300 m, we therefore also incorporate this error. For full details on UV camera procedures please see, for example^71–73^.

### Multi-GAS

Concentrations of CO_2_, SO_2_ and H_2_S were measured at 1 Hz within the volcanic plume using a miniaturised multi-component gas analyser (Multi-GAS^7,30,74^), flown on a multirotor Unoccupied Aerial System (UAS). SO_2_ and H_2_S electrochemical sensors (T3ST/F-TD2G-1A and T3H-TC4E-1A, both City Technology) are calibrated for 0–200 and 0–50 ppmv, respectively, both with an accuracy of ±2% and a resolution of 0.1 ppmv. The nondispersive infrared (NDIR) CO_2_ spectrometer (Microsensorik Smartgas Modul Premium2) is calibrated for 0–3000 ppmv with an accuracy of ±2% and a resolution of 1 ppmv. The CO_2_ spectrometer unit was wrapped in brass foil to shield the sensor board from radio frequency interference from the UAS transmission system. Pressure (±1 hPa), temperature (±0.5 °C) and relative humidity (±3%) were measured at 1 Hz using a Bluedot BME280 sensor. H_2_O concentrations were calculated according to Arden Buck equations (relating the pressure of vapour saturation to temperature for moist air; Buck, 1981) from records of temperature and relative humidity measured on-board the UAS, using a time-averaged ambient pressure of 925 mbar. Air was sampled through a 1 µm particle filter exposed to ambient air, at pump rate of 1.0 L/min. The multi-GAS was calibrated with standard references gases at the Università di Palermo one week prior to the field campaign, and again 2 weeks after. No significant sensor drift requiring data correction was identified. All sensor data were logged on-board to a micro-SD card, and also telemetered directly to the ground station where it could be visualised in real-time.

A second prototype gas sensor unit, the Aeris (Airgraph, Australia), was flown for a single flight over vent S1. SO_2_ (electrochemical) and CO_2_ (NDIR spectrometer) concentrations (together with pressure, temperature and relative humidity) were measured at a 1.25 Hz sampling rate with a repeatability of ±2% for both gas species^30^.

Gas concentration timeseries from both sensors were post-processed using MATLAB routines and Ratiocalc software^75^. CO_2_ concentrations were corrected for temperature (±0.2% full span per degree Celsius; internal compensation) and pressure (±0.15% per hPa). SO_2_ and H_2_S concentrations were corrected for reduced ambient pressure at altitude using the manufacturer-stated compensation of 0.015% and 0.008% signal per mbar, respectively. Nevertheless, barometric pressure varied by <2 mbar over the duration of the flights, as the UAS was flown at near constant altitude. Volcanogenic CO_2_ was resolved from atmospheric background by subtracting the CO_2_ concentration in ambient air (measured outside the plume where SO_2_ = 0) from the raw CO_2_ timeseries. In our measurements, H_2_S is correlated with SO_2_ at H_2_S/SO_2_ = 0.1, which is within the laboratory-determined cross-sensitivity of H_2_S to SO_2_ (13%; using standard reference gases); H_2_S is therefore considered to be below the detection limit. H_2_O exhibited the greatest temporal variability and regression uncertainties, due to varying meteorological conditions during the measurement period.

Differences in sensor response characteristics were accounted for using a deconvolution algorithm applied to the CO_2_ timeseries (Supplementary Fig. 1). The algorithm is initiated using the measured timeseries and makes use of a sensor model determined empirically from the response of the nondispersive infrared (NDIR) spectrometer to step changes in calibration gas concentration. The sensor model is best described by a windowed integral and can be thought of as an N point moving average applied to the ‘true’ input signal. Laboratory tests identified the sensor to average over ~15 seconds, hence *N* = 15 since measurements are stored at 1 Hz. The deconvolution has the effect of removing the inherent filtering effect of the sensor, hence the recovered input signal shows peaks in concentration that are steeper, narrower and often greater in amplitude than the measured signal.

Molar ratios (CO_2_/SO_2_, H_2_O/CO_2_ and CO_2_/H_2_O) were derived from gas–gas scatterplots by calculating the gradient of the best-fit linear regression line through the data. Datapoints where SO_2_ is present at <5 ppmv were excluded from the regression due to the greater error associated with very dilute plumes (e.g. Aiuppa et al., 2009). Uncertainties in derived molar gas ratios are ≥6.4% at >10 ppm SO_2_ level and 12.5% at <10 ppm SO_2_, based on the results of laboratory tests and sensor response forward modelling^30^. Uncertainties on derived CO_2_ and H_2_O volatile fluxes are based on the propagation of errors from both the molar gas ratios and the UV camera-derived SO_2_ flux timeseries, assuming a conservative uncertainty on the molar ratio at ±12%.

### Unoccupied aerial system (UAS) methods

The aerial multi-GAS was flown on-board a custom-built octocopter in the X8 configuration based on a Vulcan ‘Black Widow’ frame with hub-to-hub diameter of 120 cm (Vulcan UAV, UK). This UAS platform is described in detail by Liu et al.^30^ and in the accompanying supplementary information (Supplementary Fig. 3). Transmitted live data included information on the vehicle status, such as battery voltage and altitude, and real-time gas concentrations from the aerial multi-GAS sensor. The Aeris sensor (Airgraph, Australia) was flown on a DJI Phantom 3 quadcopter customised with a long-range transmission unit. The complete sensor package (including sensors, pump, tubing, outer casing and wireless telemetry unit) has a mass of ~300 g and has been designed specifically to integrate with the Phantom series by clipping to the struts beneath the camera gimbal as a removable modular unit^30^.

The UAS were flown from two locations in the summit area: Fortini on 07/06/2018 to access the C and S vent areas, and La Rochetta on 10/06/2018 to access the N vent area. The total duration of each flight was 15–17 min, of which ~70–80% of that time was spent within the plume. The UAS were positioned to either hover in a static position directly over each vent, or traverse through the plume a few tens of metres downwind, in order to ensure as far as possible that vent-specific emissions were measured with minimal contributions from neighbouring vents. Visual Line of Sight (VLOS) to the UAS was maintained at all times. Flight details, including target vents, are presented in Supplementary Table 1.

## Supplementary information

Supplementary Information

Description of Additional Supplementary Files

Supplementary Movie 1

## Data Availability

The data are available upon reasonable request from the authors.
