# Author Correction: Combined ground and aerial measurements resolve vent-specific gas fluxes from a multi-vent volcanic system

**DOI:** 10.1038/s41467-020-17658-8

**Published:** 2020-07-23

**Authors:** T. D. Pering, E. J. Liu, K. Wood, T. C. Wilkes, A. Aiuppa, G. Tamburello, M. Bitetto, T. Richardson, A. J. S. McGonigle

**Affiliations:** 1 Department of Geography, University of Sheffield, Sheffield, S10 2TN UK; 2 Department of Earth Sciences, University College London, London, WC1E 6BS UK; 3 Department of Aerospace Engineering, University of Bristol, Bristol, BS8 1TR UK; 4 DiSTeM, Università di Palermo, via Archirafi, 36, 90123 Palermo, Italy; 5 Istituto Nazionale di Geofisica e Vulcanologia, Sezione di Bologna, Via Donato Creti, 12, 40128 Bologna, Italy; 6 School of Geosciences, the University of Sydney, Camperdown, NSW 2006 Australia; 7 Faculty of Health, Engineering and Sciences, University of Southern Queensland, Toowoomba, QLD 4350 Australia

**Keywords:** Natural hazards, Volcanology

Correction to: *Nature Communications* 10.1038/s41467-020-16862-w; published online 16 June 2020

In the original version of this article there was an error in the Methods regarding an affiliation. This has now been corrected in the PDF and HTML versions of the article.

