# Supplementary material for: Combined ground and aerial measurements resolve vent-specific gas fluxes from a multi-vent volcano
Source: Nat Commun. 2020 Jun 16;11:3039. doi: 10.1038/s41467-020-16862-w (PMC7298010; doi:10.1038/s41467-020-16862-w)
Supplement: Supplementary file 1 — Supplementary Information [file 41467_2020_16862_MOESM1_ESM.pdf]

# Supplementary Information: Combined ground and aerial measurements resolve vent-specific gas fluxes from a multi-vent volcano

Pering et al.

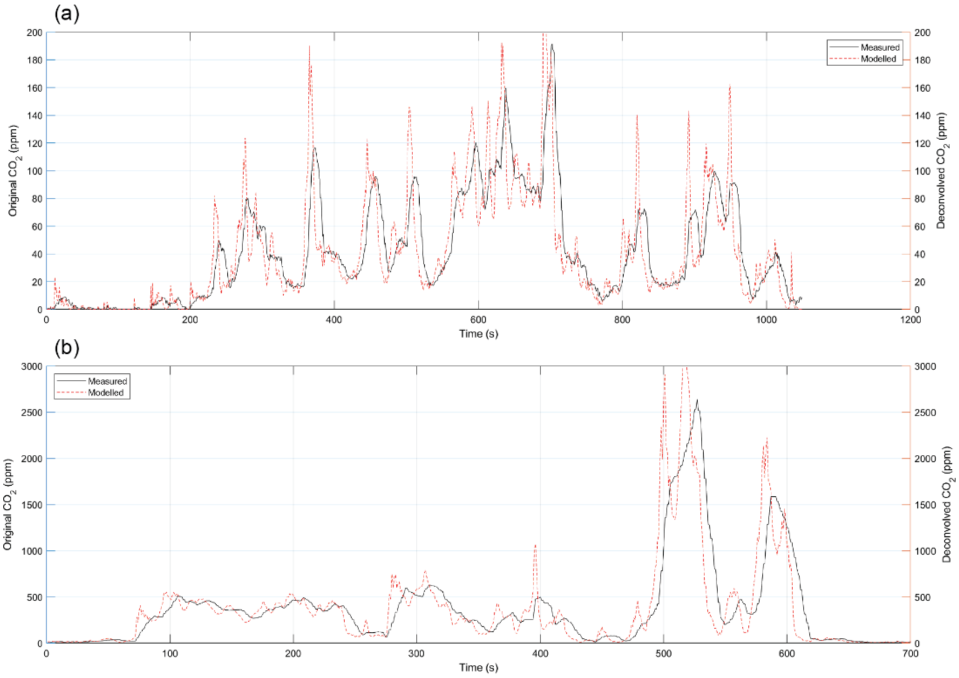


**Supplementary Figure 1**: Examples of the inverse deconvolution process applied to the $CO_2$ timeseries measured during (a) flight C[(1)] and (b) flight N2[(1)]. The deconvolved timeseries (red dashed line) is characterised by sharper peaks and shorter lags than the measured timeseries (black solid line), which is essentially filtered by the sensor response to an input signal.

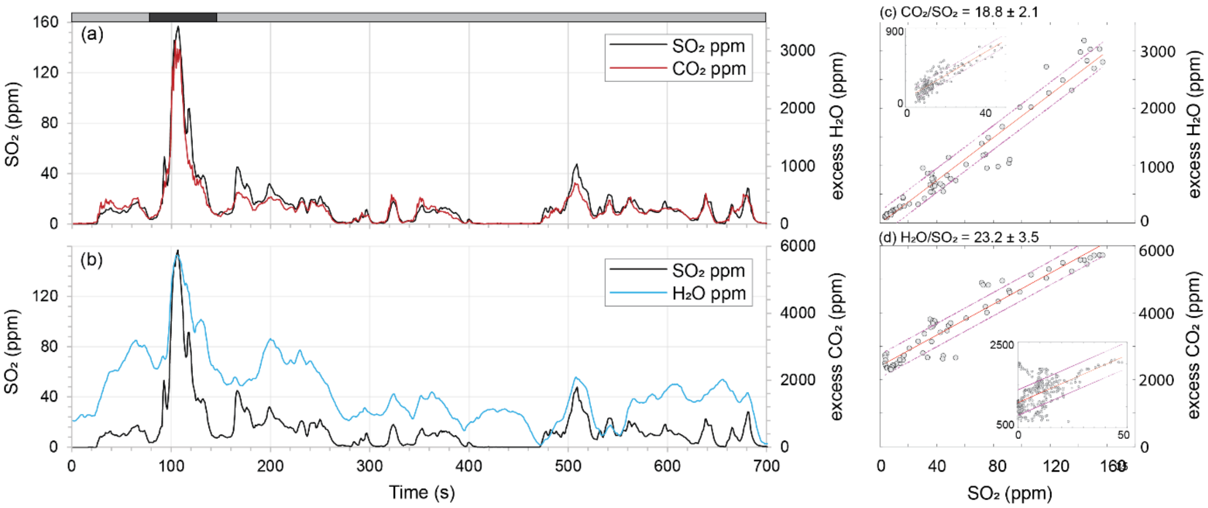


**Supplementary Figure 2:** Molar gas composition data for an UAS overflight (N2[(2)]) on 10/06/18 during passive degassing and a Strombolian explosion from vent N2, showing gas concentration timeseries for (a) $SO_2$ and $CO_2$, (b) $SO_2$ and $H_2O$. The shaded band at the top indicates passive (grey) and explosive (black) phases. (c, d) Scatterplots for the explosive phase emissions of $SO_2$-$CO_2$ and $SO_2$-$H_2O$ respectively, displaying the least squares regression and 95% confidence intervals. Gas molar ratios correspond to the gradient of the regression line shown. Inset scatterplots show data for the passive degassing phase.

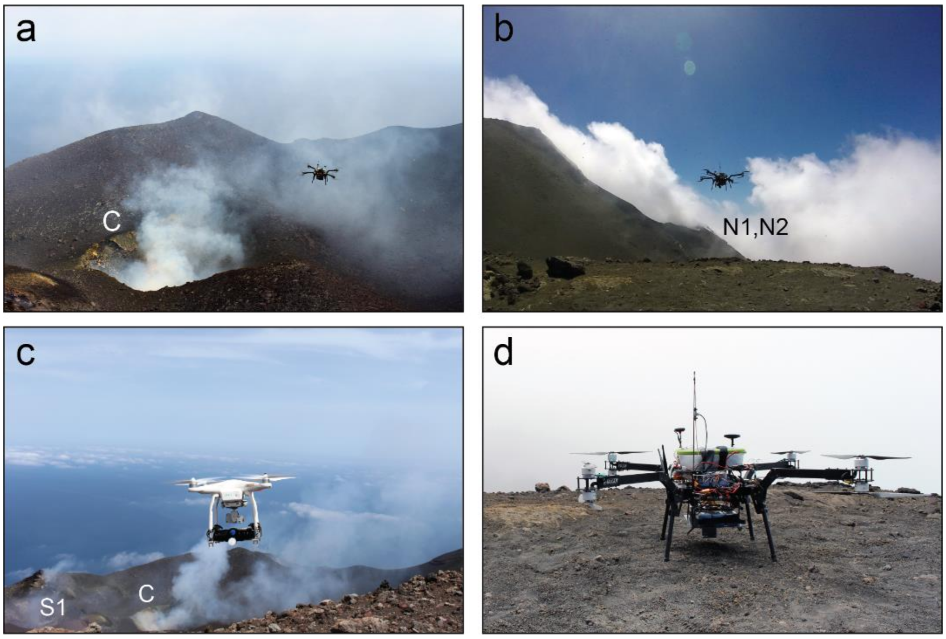


**Supplementary Figure 3:** Unoccupied Aerial Systems (UAS) used in this study. (a, b, d) Multi-GAS sensor in upward-mounting position on a Vulcan Black Widow X8 (Vulcan UAV, UK) platform with custom avionics. (c) Aeris (Airgraph, Australia) mounted on a DJI Phantom 3 Pro quadcopter. Takeoff and landing sites included (a, c) Fortini – southwestern rim of crater terrace, and (b, d) La Rochetta – northern rim of crater terrace.

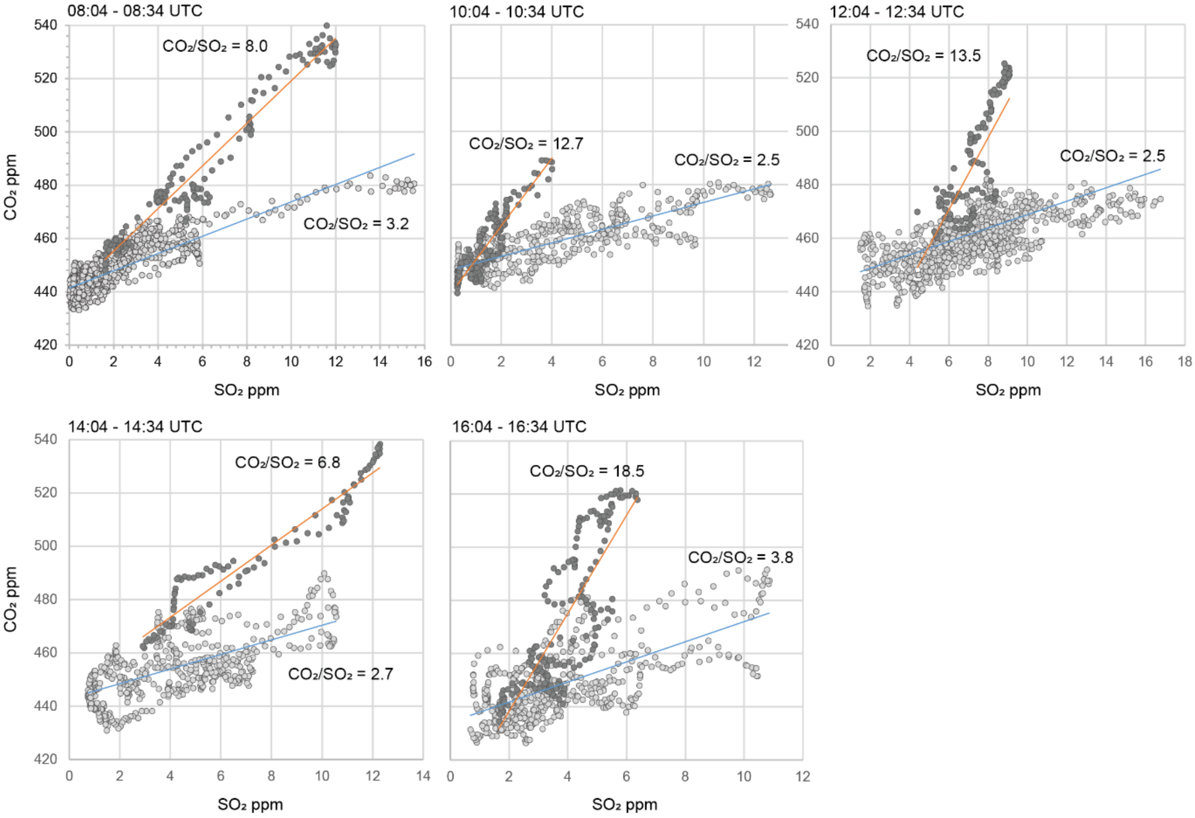


**Supplementary Figure 4:** Ground-based multi-GAS measurements from the permanent monitoring station located on the crater rim of Stromboli. Data shown for 07/06/2018. Measurements are automatically acquired for 30 minutes every 2 hours. Molar ratios are only calculated for time intervals where $SO_2$ concentrations exceed 5 ppm. Passive degassing (light grey symbols) represents persistent activity measured over tens of minutes, whilst explosive degassing (dark grey symbols) represents transient peaks lasting <60 s.

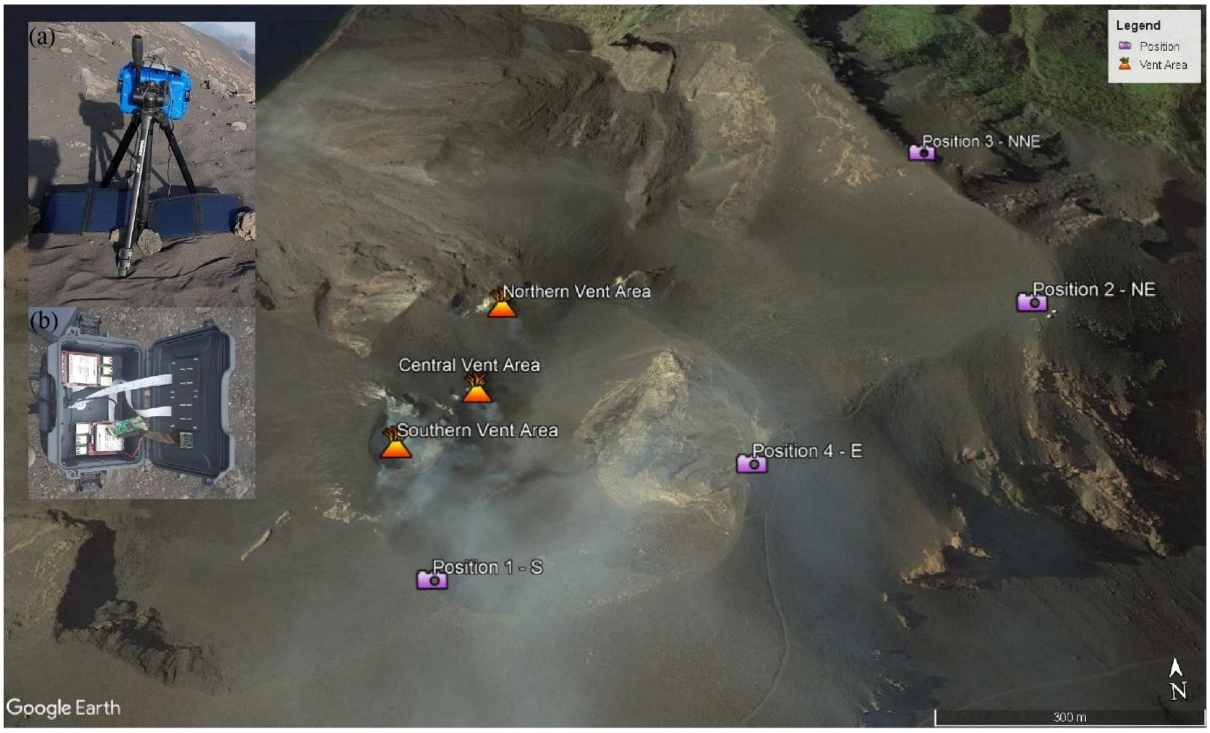


**Supplementary Figure 5:** Map of the summit area including camera positions and approximate locations with respect to the vent area. Vent areas are also indicated on the image. Inset are images of the UV camera setup, in (a) the UV camera in Position 2 with solar panels attached, and (b) the interior of the camera showing attached PiJuice, mobile phone batteries, and a Raspberry Pi Zero which operated as an additional time-lapse visible camera.

**Supplementary Table 1**

| Flight name | Date and time | Start time (UTC) | Payload | Target vent | Duration (time in plume), mins |
|---|---|---|---|---|---|
| S1 | 04/06/2018 | 08:35 – 08:45 | Aeris | S1 | 10 (5.5) |
| C(1) | 07/06/2013 | 07:15 – 07:32 | Multi-GAS | C | 17 (13) |
| C(2) | 07/06/2018 | 08:07 – 08:25 | Multi-GAS | C | 18 (5) |
| N2(1) | 10/06/2018 | 10:03 – 10:15 | Multi-GAS | N2 | 18 (9.5) |
| N2(2) | 10/06/2018 | 11:16 – 11:32 | Multi-GAS | N2 | 16 (11) |
