# Supplementary material for: Combined ground and aerial measurements resolve vent-specific gas fluxes from a multi-vent volcano
Source: Nat Commun. 2020 Jun 16;11:3039. doi: 10.1038/s41467-020-16862-w (PMC7298010; doi:10.1038/s41467-020-16862-w)
Supplement: Supplementary file 2 — Description of Additional Supplementary Files [file 41467_2020_16862_MOESM2_ESM.pdf]

Description of Additional Supplementary Files

Supplementary Movie 1

Video footage acquired with an UAS, which includes an overflight of the whole summit area; footage during a strombolian explosion from vent S1, where explosive gas release at one vent is preceded by a ~1 s doming at a second vent in close proximity; and a close-up of puffing at vent area C.
